# Primary Health Care assessment in the COVID-19 pandemic from physicians’ and nurses’ perspective

**DOI:** 10.1590/0034-7167-2022-0475

**Published:** 2023-07-31

**Authors:** Paula dos Santos Brito, Lívia Maia Pascoal, Mário Vinicius Teles Costa, Lorrany Fontenele Moraes da Silva, Liana Priscilla Lima de Melo, Marcelino Santos, Francisca Elisângela Teixeira Lima, Floriacy Stabnow Santos

**Affiliations:** I Universidade Federal do Maranhão. Imperatriz, Maranhão, Brazil; II Universidade Federal do Maranhão. São Luís, Maranhão, Brazil; III Universidade Federal do Ceará. Fortaleza, Ceará, Brazil

**Keywords:** COVID-19, Primary Health Care, Health Personnel, Health Care Evaluation Mechanisms, Health Services Research, COVID-19, Atención Primaria de Salud, Profesionales de la Salud, Personal de Salud, Investigación Sobre Servicios de Salud, COVID-19, Atenção Primária à Saúde, Profissionais da Saúde, Avaliação de Serviços de Saúde, Pesquisa de Serviços de Saúde

## Abstract

**Objectives:**

to assess the operationalization of Primary Health Care in the COVID-19 pandemic, according to Primary Care Assessment Tool: PCATool-*Brasil* attributes, from physicians’ and nurses’ perspective.

**Methods:**

a cross-sectional study, carried out with 99 physicians and nurses from Basic Health Units in a state in northeastern Brazil, with the aid of the adapted instrument PCATool-*Brasil*.

**Results:**

Essential Score was classified as high performance (6.6) and General Score as low performance (6.5). First Contact Access, Care Integration, and Community Guidance scores were <6.6. The best performances were attributed to the Longitudinality, Comprehensiveness and Family Guidance services (scores>6.6).

**Conclusions:**

the attributes of Primary Health Care, in general, showed values above or close to the cut-off point in the assessment. These data can support strategies for local and national managers to strengthen Primary Health Care in the COVID-19 pandemic and future public health emergencies.

## RESUMO

**Objetivos:**

avaliar a operacionalização da Atenção Primária à Saúde na pandemia de COVID-19, segundo os atributos do *Primary Care Assessment Tool: PCATool*-Brasil, na perspectiva de médicos e enfermeiros.

**Métodos:**

estudo transversal, realizado com 99 médicos e enfermeiros de Unidades Básica de Saúde de um estado do Nordeste do Brasil, com auxílio do instrumento adaptado PCATool-Brasil.

**Resultados:**

Escore Essencial foi classificado como alto desempenho (6,6), e Escore Geral, como baixo desempenho (6,5). Os escores de Acesso de Primeiro Contato, Integração do Cuidado e Orientação Comunitária foram <6,6. Os melhores desempenhos foram atribuídos aos serviços Longitudinalidade, Integralidade e Orientação Familiar (escores > 6,6).

**Conclusões:**

os atributos da Atenção Primária à Saúde, em geral, apresentaram valores superiores ou próximos ao ponto de corte na avaliação. Esses dados podem subsidiar estratégias de gestores locais e nacionais para o fortalecimento da Atenção Primária à Saúde na pandemia de COVID-19 e futuras emergências de saúde pública.

## RESUMEN

**Objetivos:**

evaluar la operacionalización de la Atención Primaria de Salud en la pandemia de la COVID-19, según los atributos del Instrumento de Evaluación de la Atención Primaria: PCATool-Brasil, en la perspectiva de médicos y enfermeros.

**Métodos:**

estudo transversal, realizado com 99 médicos e enfermeiros de Unidades Básicas de Saúde de um estado do Nordeste do Brasil, com auxílio do instrumento adaptado PCATool-Brasil.

**Resultados:**

Escore Essencial foi classificado como alto desempenho (6,6), e Escore Geral, como baixo desempenho (6,5). Las puntuaciones de acceso al primer contacto, integración de la atención y orientación comunitaria fueron <6,6. Los mejores desempeños fueron atribuidos a los servicios de Longitudinalidad, Integralidad y Orientación Familiar (puntuaciones > 6,6).

**Conclusiones:**

los atributos de la Atención Primaria de Salud, en general, presentaron valores superiores o cercanos al punto de corte en la evaluación. Estos datos pueden respaldar estrategias para que los administradores locales y nacionales fortalezcan la Atención Primaria de Salud en la pandemia de COVID-19 y futuras emergencias de salud pública.

## INTRODUCTION

The public health emergency caused by the Severe Acute Respiratory Syndrome-related Coronavirus-2 (SARS-CoV-2) pandemic is the causative agent of the disease called coronavirus disease (COVID-19). Due to the speed of transmission of the infection, illness, need for social distancing and hospital care, it affected people’s lives and exposed the weaknesses of health systems globally, including those in Brazil. In the Brazilian scenario, the first COVID-19 case was confirmed on February 26, 2020, followed by community transmission throughout the national territory, and the first death occurred on March 17 in the city of São Paulo^(1)^.

Among the regions of the country with the highest number of cases, the Southeast (12,249,294), South (6,761,049) and Northeast (6,288,380) stand out. Maranhão is the sixth state in the Northeast region with the highest number of COVID-19 cases^(2)^. According to the Epidemiological Bulletin of the State Department of Health of Maranhão, until August 10, 2022, the state recorded 464,645 cases and 10,960 deaths from the disease, and the highest numbers of cases and deaths are in the capital São Luís (72,925 cases and 2,681 deaths) and in the municipality of Imperatriz (25,516 cases and 907 deaths)^(3)^.

Studies have shown that most people with COVID-19 have mild or asymptomatic infections^(4, 5)^. Primary Health Care (PHC) has been prioritized in the care of these cases, in Basic Health Services, and only the most serious cases are referred to the Urgency and Emergency Network^(5)^. The decentralization of Basic Health Units (BHU) contributes to people with mild and moderate cases seeking the basic network as the first access in the search for care^(6)^.

During the pandemic caused by COVID-19, PHC plays a fundamental role in responding to the population’s assistance by offering resolute care and early identification of serious cases that must be managed in specialized services^(5, 7)^. In view of this, the Ministry of Health (MoH) has structured protocols that include telecare, severity stratification flowchart, clinical management and funding for municipalities to extend the opening hours of BHU and Family Health Strategy (FHS) through the *Saúde na Hora* Program^(8)^.

Considering the context of COVID-19 in the state of Maranhão, BHU, among them those of São Luís and Imperatriz, were organized for assistance with flu and respiratory syndromes, in addition to other measures for disease management and control^(8, 9)^. Given the situation caused by the COVID-19 pandemic, it is essential to identify the PHC response in meeting the challenges imposed to assist the population. Thus, assessing the actions and policies implemented in the health system is important to measure the effectiveness and progress of the actions carried out^(10)^.

Among the reference models recommended by the MoH to assess PHC care quality, the Primary Care Assessment Tool (PCA-Tool) stands out as effective and internationally recommended for planning and assessing quality in health care, with versions for assessment from adults’, children’s, health professionals’ and managers’ perspectives. PCATool stands out due to the lack of other validated instruments with high methodological rigor to assess the presence and extent of PHC essential and derived attributes^(11)^.

The indicators that make up PCATool assess the extent of affiliation with the health service, covering the First Contact Access (accessibility), Longitudinality, Coordination (Care Integration and information system), Comprehensiveness (services available and services provided) essential attributes, and also the Family and Community Guidance derived attributes^(11)^. PCATool has been used to assess the operationalization of PHC through the analysis of its attributes in several studies in the health area, adapting contexts and scenarios of interest such as in Brazil, Chile and Spain^(11, 12, 13, 14)^.

Therefore, it is important to know the factors that interfere with the performance of PHC attributes in the context of the COVID-19 pandemic, as PHC analysis, based on PCATool, allows the assessment within a real context of public emergency and can contribute to gather information about the phenomenon, listening to the different interested agents, in the elaboration of a management plan for future emergencies, safeguarded by methodological rigor and already internationally recognized and validated.

The relevance of assessing the operationalization of PHC, considering the current COVID-19 pandemic and future emergencies, contributes to scientific production by representing public health policy practices in general PHC structure and organization in the local system. Furthermore, assessing the performance of PHC attributes in coping with COVID-19 is imperative in preparing health system responses to combat the disease and its consequences.

## OBJECTIVES

To assess the operationalization of Primary Health Care in the face of the COVID-19 pandemic, according to PCATool-Brasil attributes, from physician’ and nurses’ perspective.

## METHODS

### Ethical aspects

In compliance with the ethical precepts of research, this study was approved by the Research Ethics Committee (REC) of the *Universidade Federal do Maranhão* (UFMA).

### Study design, period, and place

This is a cross-sectional study, carried out from December 2020 to November 2021 with physicians and nurses from the city of Imperatriz and capital São Luís, located in the state of Maranhão, guided by the EQUATOR network Strengthening the Reporting of Observational Studies in Epidemiology (STROBE). According to 2021 estimates by the Brazilian Institute of Geography and Statistics (IBGE), São Luís and Imperatriz have a population of 1,115,932 and 259,980 inhabitants, respectively^(15)^. They are structured with 1,944 BHU, composed of 2,282 FHS teams, in São Luís, with 110 FHS in 63 BHU, and in Imperatriz, with 62 FHS teams in 33 units^(16)^.

### Population or sample; inclusion and exclusion criteria

The population consisted of medical professionals and nurses working in BHU in coping with the COVID-19 pandemic. Participants were selected according to an intentional snowball sample^(17)^. Professionals who treated suspected or confirmed COVID-19 cases in PHC for a period equal to or greater than six months were included. Professionals who were absent from the health unit due to health problems or vacations at the time of data collection were excluded.

### Study protocol

For data collection, the PHC Primary Care Assessment Tool (PCATool), Brazil, professional version^(11)^ and a structured instrument with sociodemographic variables (gender, professional category, title, age, time since professional training, time working in primary care) were adapted. PCATool is composed of PHC’s essential and derived attributes. Essential attributes are First Contact Access, Longitudinality, Coordination (Care Integration), Comprehensiveness (services available and services provided), and derived attributes are Family Guidance and Community Guidance^(11)^.

In this research, the contents of these attributes were adjusted to assess the health service in the context of COVID-19, based on MoH manuals and protocols related to the theme^(4-5)^, and included the essential and derived attributes from the PCATool-Brasil instrument, for physicians and nurses extended version. The instrument was submitted to a group of 16 specialists, which consisted of teaching nurses with experience in PHC and nurses working in PHC, who assessed the format, concepts and items, regarding the criteria of relevance, clarity and precision^(18)^. After making the adjustments suggested by the experts, the final version of the instrument was used for data collection.

The instrument had six domains and 62 items that were organized into a semi-structured and self-administered questionnaire. Each attribute’s assessment was obtained using a Likert-type scale from 1 to 4 points ((1 = definitely not, 2 = probably not, 3 = probably yes and 4 = definitely yes), with the addition of option 9 (do not know/do not remember)^(11)^.

Due to the context of the COVID-19 pandemic and the adoption of security measures, data collection was carried out, as a priority, by interview via telephone contact or made available online via email or WhatsApp according to participants’ availability. The Primary Care coordination of the municipalities where the research was carried out communicated professionals about the study and provided their telephone contacts.

In order to capture a greater number of professionals working in PHC, active face-to-face searches were carried out in the basic units of the investigation scenario and the PHC professionals themselves informed names, e-mails and contacts of new participants who were invited and included in the research. Moreover, the survey was disseminated in a group on a social network (WhatsApp), made up of all FHS registered nurses in the municipalities of Imperatriz and São Luís, in an attempt to attract a larger number of professionals.

Data collection was carried out by a single researcher, who had a degree in nursing, which could be done in two ways, according to professionals’ preference: interview via telephone contact, in which the researcher filled out the collection instrument, whose average duration was 20 minutes; or through a questionnaire made available online, on a social network or by email, with the help of Google Forms. In the latter case, a period of 15 days was established for returning the completed questionnaire. The Informed Consent Form was obtained from all research participants online.

Initially, the number of physicians and nurses was estimated based on the number of BHU and FHS in the two municipalities to compose the sample. However, due to the high workload imposed by the pandemic and the absence of professionals due to having contracted COVID-19, it was decided to include in the research all physicians and nurses who met the established inclusion criteria and agreed to participate in the study, through a convenience sampling process.

### Analysis of results, and statistics

The collected data were organized in Microsoft Excel 2010 and analyzed in the Statistical Package for the Social Sciences (SPSS), version 24.0. In the univariate descriptive analysis, measures of absolute frequency, percentages and 95% confidence intervals, central tendency (means) and dispersion (standard deviation) were used. The results were organized in tables.

Each attribute’s assessment was obtained following the guidelines of PCATool-Brasil itself. From the answers obtained, the average score of each attribute was calculated, by the sum of the answers of each item, divided by the total number of items of each essential and derived attribute. When option 9 (do not know/do not remember) were greater than 50% or more of the total items in the component, the score was not calculated for that interviewed professional, leaving it blank (“missing”) in the database. When less than 50% of total items in the component, value “9” was transformed into value “2” (“probably not”)^(11)^.

Each essential attribute of PCATool-Brasil is formed by a component related to the structure and another to the care process. The general score was calculated from the sum of essential and derived scores divided by the total number of components. Subsequently, these scores were transformed into a scale from 0 to 10 to verify the service effectiveness using the following formula: (score obtained - 1) x 10/3. The value obtained was classified as high (≥ 6.6) or low (< 6.6) performance. The score cut-off point ≥ 6.6 refers to at least response category 3 (probably yes) of the instrument’s items^(11)^.

## RESULTS

A total of 99 medical health professionals (23) and nurses (76) who work with suspected or confirmed COVID-19 cases at BHU in Imperatriz and São Luís participated in this research. In Table 1, it can be observed that, in general, the sample was predominantly composed of women (81.82%), nurses (76.77%) and with a specialist degree (82.83%). The mean age was 35 years, with a minimum of 23 and a maximum of 55 years. The average time of professional training was 6 years, with an average time working in PHC of 4 years. Most medical professionals (73.91%) and a significant portion of nurses (42.11%) had another employment relationship.

**Table 1:** Profile of health professionals, physicians and nurses working in the COVID-19 pandemic in the Basic Health Units of the municipalities of Imperatriz and São Luís, Maranhão, Brazil (N = 99)

Variables	n	%
Sex		
Female	81	81.82
Male	18	18.18
Professional category		
Nurse	76	76.77
Physician	23	23.23
Title		
Specialist	82	82.83
Master’s or doctoral degree	1	1.01
Not informed	16	16.16

	Minimum	Maximum	Mean	SD*
Age (years)	23	55	35.04	6.96
Time of professional training (years)	01	35	6.81	5.38
Time working in Primary Health Care (years)	01	32	4.32	4.32

*SD - standard deviation.

In Table 2, the scores of PHC’s essential and derived attributes in the assessment of medical and nursing health professionals can be observed. Among the attributes considered essential, First Contact Access had the lowest general mean (5.22 ± 0.97). In derived attributes, only Family Guidance achieved high performance (6.85 ± 0.96). Community Guidance had low performance in both research scenarios (5.55 ± 1.34). The attributes with the best performances were Longitudinality (6.72 ± 1.00) and Comprehensiveness (7.13 ± 0.99) and Family Guidance (6.85 ± 0.96).

**Table 2:** Scores attributed by physicians and nurses working in the COVID-19 pandemic in Basic Health Units in the municipalities of Imperatriz and São Luís, Maranhão, Brazil, according to the essential and derived attributes of PCATool-*Brasil*, professional version (N = 99)

Scores	Investigated scenarios					Imperatriz					São Luís				
	X̅ ± SD* (95% CI^§^)	Low score < 6.6		High score ≥ 6.6		X̅ ± SD* (95% CI^§^)	Low score < 6.6		High score ≥ 6.6		X̅ ± SD* (95% CI^§^)	Low score < 6.6		High score ≥ 6.6	
		n	%	n	%		n	%	n	%		n	%	n	%
First Contact Access	5.22 ± 0.97 (5.00-5.39)	91	91.92	8	8.08	5.10 ± 1.00 (4.83-5.37)	52	92.86	4	7.14	5.32 ± 0.93 (5.03-5.61)	39	90.70	4	9.30
Longitudinality	6.72 ± 1.00 (6.52-6.92)	21	21.21	78	78.79	6.76 ± 1.72 (6.45-7.06)	15	26.79	41	73.21	6.68 ± 0.82 (6.43-6.94)	6	13.95	37	86.05
Care Coordination	6.89 ± 1.28 (6.64-7.15)	27	27.27	72	72.73	7.21 ± 1.23 (6.88-7.54)	7	12.50	49	87.50	6.48 ± 1.24 (6.10-6.86)	20	46.51	23	53.49
*Care Integration*	6.44 ± 1.05 (6.23-6.65)	34	34.34	65	65.66	6.52 ± 1.00 (6.25-6.80)	19	33.93	37	66.07	6.33 ± 1.00 (5.99-6.67)	15	34.88	28	65.12
*Information system*	7.35 ± 1.86 (6.97-7.72)	15	15.15	84	84.85	7.89 ± 1.87 (7.39-7.40)	6	10.71	50	89.29	6.64 ± 1.61 (6.14-7.13)	9	20.93	34	79.07
Comprehensiveness	7.13 ± 0.99 (6.93-7.33)	28	28.28	71	71.72	7.41 ± 1.12 (7.11-7.71)	11	19.64	45	80.36	6.76 ± 0.63 (6.57-6.96)	17	39.53	26	60.47
*Available Services*	7.19 ± 1.00 (6.99-7.39)	8	8.08	91	91.92	7.46 ± 1.12 (7.11-7.71)	6	10.71	50	89.29	6.84 ± 0.55 (6.67-7.01)	2	4.65	41	95.35
*Services Provided*	7.07 ± 1.17 (6.84-7.30)	32	32.32	67	67.68	7.37 ± 1.28 (7.02-7.71)	15	26.79	41	73.21	6.68 ± 0.30 (6.41-6.95)	17	39.53	26	60.47
Family Guidance	6.85 ± 0.96 (6.66-7.05)	28	28.28	71	71.72	6.99 ± 1.15 (6.68-7.30)	18	32.14	38	67.86	6.68 ± 0.60 (6.49-6.86)	10	23.26	33	76.74
Community Guidance	5.55 ± 1.34 (5.28-5.81)	73	73.74	26	26.26	5.80 ± 1.26 (5.46-6.14)	36	64.29	20	35.71	5.22 ± 1.15 (4.79-5.65)	37	86.05	6	13.95

X̅ Mean; *SD - standard deviation; §IC95% - 95% confidence interval.

In Table 3, the Essential Score and General Score scores can be observed. In general, the Essential Score of the two municipalities was of high performance (6.66 ± 0.81), and the General Score, of low performance (6.54 ± 0.78). Only the municipality of Imperatriz reached a high performance assessment in both scores, with values very close to the cut-off line (Essential: 6.85 ± 0.86; General: 6.73 ± 0.81). However, it is noteworthy that, in the two investigated scenarios, there were attributes assessed as low performance, with scores of five, on average.

**Table 3:** Scores attributed by physicians and nurses working in the COVID-19 pandemic in Basic Health Units in the municipalities of Imperatriz and São Luís, Maranhão, Brazil, according to the Essential and General Scores of PCATool-*Brasil*, professional version (N = 99)

Scores	Investigated scenarios					Imperatriz					São Luís				
	X̅ ± SD* (95% CI^§^)	Low score < 6.6		High score ≥ 6.6		X̅ ± SD* (95% CI^§^)	Low score < 6.6		High score ≥ 6.6		X̅ ± SD* (95% CI^§^)	Low score < 6.6		High score ≥ 6.6	
		n	%	n	%		n	%	n	%		n	%	n	%
Essential Score	6.66 ± 0.81 (6.50-6.82)	55	55.56	44	44.44	6.85 ± 0.86 (6.62-7.08)	25	44.64	31	55.36	6.41 ± 0.67 (6.21-6.62)	30	69.77	13	30.23
General Score	6.54 ± 0.78 (6.39-6.70)	57	57.58	42	42.42	6.73 ± 0.81 (6.52-6.95)	25	44.64	31	55.36	6.30 ± 0.66 (6.09-6.50)	32	74.42	11	25.58

X̅ Mean; *SD - standard deviation; §IC95% - 95% confidence interval.

## DISCUSSION

PHC assessment from professionals’ perspective, in general, obtained values above or close to the cut-off point, which corroborates with studies carried out with the PCATool before the COVID-19 pandemic, in which PHC quality was assessed from the perspective of physicians and nurses working at BHU in the states of Rio Grande do Sul^(19)^, whose results were 6.66 for essential attributes and 7.60 for derivatives, and in Goiás^(20)^ with values of 7.68 and 9.11, respectively.

In the context of public health, service assessment stands out as one of the best mechanisms to respond to the population’s demands, to assess the performance of the service offered with regard to precepts of universality, accessibility, quality of care and others, in a scenario of epidemiological changes and economic crises^(21)^. A high performance assessment indicates a solid PHC, whose disease prevention, health promotion and recovery actions are developed in a resolute, universal and equitably distributed manner^(22)^.

The First Contact Access, Care Coordination in the Care Integration component and Community Guidance attributes obtained scores attributed as low performance in the two investigated scenarios. Regarding the First Contact Access attribute, which in this study had the worst performance, a low performance classification was also identified in several Brazilian studies when assessed from health professionals’ perspective^(23, 24, 25)^.

The low score attributed to accessibility may be related to the fact that most FHS teams in the investigated scenario work only on weekdays, with appointments scheduled in advance. Despite extended hours in the *Saúde na Hora* Program (8 a.m. to 8 p.m.), the restricted hours in the night period may not cover the needs of users of the assigned area who work during business hours, considering the time to travel from the workplace. Another limiting factor to access is the unavailability of means of communication with patients at night or on weekends, especially in acute cases. This is a national scenario, a factor that results in a search for emergency care services and weakens users’ bond with PHC services^(25)^.

Low performance assessment suggests failure in continuity of organizational situations that facilitate accessibility in PHC, since the items assessed in the PCATool instrument for the First Contact Access attribute refer to appointment scheduling, offer of services with extended hours and outside working hours, waiting time, availability of a telephone to contact the service and health professionals, among others. Access is essential for users to be able to reach services and receive first-contact assistance and must be considered by municipal managers for the organization of services offered for this level of care^(26)^.

The Longitudinality attribute, despite being classified with high performance, presented scores very close to the cut-off point in Imperatriz (6.76) and São Luís (6.68). A similar result was found in the study on Longitudinality assessment in BHU using the PCATool in Rio Verde, Goiás, with a score of 6.77^(27)^. This assessment demonstrates a good interpersonal relationship between professionals and users with regard to knowledge about their previous health conditions, difficulty in acquiring medication due to socioeconomic situations and enough consultation time to express their doubts^(11)^.

In the COVID-19 pandemic, this assessment positively corroborates case management, despite the challenges posed by the high demand for care and the epidemiological uncertainties of the disease. In this regard, the importance of a bond with health professionals in follow-up and continuity of care in accountability between professionals and users over time is recognized^(28)^. It should be noted that, with the centralization of care for patients with COVID-19 in specific BHU, care was not restricted to the coverage area, which could interfere with the assessment and justify the result close to the cut-off point.

Care Coordination in the Care Integration component was assessed as low performance by professionals. In Brazil, PHC, through FHS, included the creation of specialty services in health districts, implementation of a regulatory system and electronic medical records, management and clinical protocols, communication initiatives and matrix support through the integration of Health Care Networks, aiming to improve the Care Coordination attribute^(29)^.

These results show that there is no assessment of results of consultations carried out outside the basic unit by health professionals and highlights the need for adjustments in the follow-up of referral to referral and counter-referral. Studies carried out before the COVID-19 pandemic also identified weaknesses with regard to the Care Integration component^(23, 30)^. In order to achieve high performance, Care Coordination needs to guarantee continuity of care at other service points, through Care Integration and use of information systems^(31)^.

The Comprehensiveness attribute obtained a high performance in the two scenarios studied, evidencing PHC’s good structural capacity in offering the services available and provided for the care of COVID-19 cases. It was assessed that physicians and nurses carried out actions of guidance, monitoring and reassessment of cases through communication technologies, such as calls and video calls, which allowed for continuity in service provision and user monitoring, in addition to the provision of services such as vaccines, support groups and referrals to other reference services.

On the international scene, the PHC structure has been identified as a limiting factor in facing the COVID-19 pandemic, due to the fact that the PHC has few communication technologies, as low availability of computers with internet access in BHU for continuity of services provided^(32)^. The opposite situation was identified in this study, since, in the assessment of physicians and nurses, the units had the structure to continue offering the services available in the unit during the COVID-19 cases.

In the assessment of derived attributes, Family Guidance was assessed with high performance, with a score very close to the cut-off line in the two municipalities assessed. Another research^(33)^ that assessed PHC in São Paulo from the perspective of 102 health professionals, also obtained a high performance result and the score obtained was 8.53. Family Guidance allows the involvement of physicians and nurses with families, contributing to bond formation. Living together allows professionals to have a broad view of family’s needs and priorities, facilitating the development of an adequate care plan. Thus, when knowing the family configuration, professionals can provide individualized and assertive guidance in care, given the various sociodemographic and economic contexts that can configure family arrangements^(34)^.

The Community Guidance attribute assessment had the second worst performance in this study. This result indicates the low community performance of these professionals in listening to users in COVID-19 cases, reflecting the need for the multidis-ciplinary team that makes up FHS to act as physicians, nurses and community health workers in the territorial action in space use and community collaboration in the identification of cases, in addition to effective monitoring strategies.

It is through contact between health professionals and the community that it is possible to collect epidemiological data and, consequently, obtain more resources and improve the planning of strategic actions for PHC services. Some factors seem to be related to better implement the Community Guidance attribute in PHC, such as home visits carried out by all professionals, as it facilitates health surveillance, listening to the needs and monitoring of families in the community^(35)^.

Furthermore, it is necessary to consider that people’s health needs occur within a social context and knowledge of this reality by primary care teams is essential for planning and organizing strategic actions of PHC services^(34)^. In the scenario of COVID-19, this attribute becomes fundamental in monitoring and adhering to care practices. In the BHU context, this attribute was expected to present higher scores, since this strategy is based on user participation and social control^(12)^.

The Essential Score was classified as high performance, however, the value obtained was close to the cut-off point (6.66) and the best results were identified in the municipality of Imperatriz (6.85). The General Score was assessed as low performance, similar to other studies carried out in different scenarios and outside the context of COVID-19, which also showed limited performance of attributes^(22, 25)^. This result demonstrates that PHC has shown, over the years, weaknesses in terms of the operationalization of its attributes. In view of this, the importance of reorganizing the work process in PHC, in the face of the COVID-19 pandemic, is reinforced in order to preserve the attributes First Contact Access, Comprehensiveness, Longitudinality, Coordination of Care, Family Guidance and Community Guidance.

### Study limitations

As limitations of this study, the use of convenience sampling stands out, since data collection was carried out upon availability and acceptance in an environment of high work demand and challenges imposed by the pandemic in the scenario investigated. Due to the need for physical distancing due to COVID-19, access to attracting participants was limited, such as performing phone contacts, successful phone calls, which sometimes did not complete the call, or calls were not answered.

Another point to be considered is that, although the results represent the two scenarios of the state of Maranhão with a high number of COVID-19 cases, these results cannot be generalized. Cross-sectional studies have limitations on the temporal relationship, which, in the scenario of PHC and the COVID-19 pandemic, data may represent a moment from professionals’ perspective on PHC performance, since the pandemic is still ongoing.

### Contributions to nursing, health, and public policies

This study can support managers to reorganize the PHC work process, highlighting public health emergency situations such as the one caused by the COVID-19 pandemic. It also contributes to management and care practice, as it identified evidence of the performance of the current care provided, little explored about PHC and unprecedented in assessing the attributes in the scenario of COVID-19. It can guide decision-making by health professionals in BHU, since nurses act in guiding the nursing team, users and the community.

## CONCLUSIONS

The PHC Essential Score was classified as high performance (6.6) and the General Score was low performance (6.5). The First Contact Access, Care Integration and Community Guidance attributes performed poorly, with lower scores < 6.6. The attributes with the highest performances were Comprehensiveness and Care Coordination in the Information System component, with scores ≥ 6.6 in the two scenarios investigated. Despite these results, the effort of local managers in organizing services to care for COVID-19 cases is recognized.

The results obtained can support actions in the planning and improvement of strategies by local and national managers in the search for public policies to strengthen PHC in the face of the COVID-19 pandemic and future public health emergencies. The innovative potential of the study carried out in a context of overload of professionals and health services caused by the high demand for COVID-19 cases is highlighted, making it possible to assess PHC in the scenario investigated, which can contribute to improvements in the attributes that obtained low scores and solidify the actions already undertaken in the attributes classified as high performance.
